# Dataset of focus prosody in Japanese phone numbers

**DOI:** 10.1016/j.dib.2019.104139

**Published:** 2019-06-11

**Authors:** Yong-cheol Lee, Satoshi Nambu, Sunghye Cho

**Affiliations:** a Cheongju University, South Korea; b Monash University, Australia; c University of Pennsylvania, USA

**Keywords:** Bipodic template, Focus prosody, Japanese, Phone numbers

## Abstract

The data in this article present position-dependent variation of focus prosody within phone number strings in Tokyo Japanese. Four acoustic parameters (duration, mean intensity, maximum pitch, and time-normalized pitch contours) are reported to illustrate focus prosody of Japanese phone numbers, separately for broad focus and corrective focus. The data also include four attached files: 1) time-normalized pitch contours for all speakers (Appendix A), 2) aggregated data of duration, mean intensity, and maximum pitch for on-focus effects (Appendix B), 3) a Python script automatically generating target stimuli (Appendix C), and 4) target stimuli used for each focus type (Appendix D). The data set can be used for several research projects including speech recognition, focus study, speaker variation in marking prosodic focus, and prosody modeling in Tokyo Japanese. Detailed discussion of data interpretation can be found in the article entitled “Prosodic focus of telephone numbers in Tokyo Japanese” (Lee et al.).

**Table:** Specifications Table

Subject area	*Linguistics*
More specific subject area	*Phonetics*
Type of data	*Figure, spreadsheet*
How data was acquired	*Acoustic measurements from speech recordings done in a lab setting*
Data format	*Figures, CSV files*
Experimental factors	*Two main experimental factors: focus type (broad vs. corrective); focus position within a bipodic template (first vs. second)*
Experimental features	*A mismatch in focus prosody depending on the position with or without an accentual peak in phone number strings*
Data source location	*Cheongju University, Seoul, Korea*
Data accessibility	*Data within the article*
Related research article	*Lee, Yong-cheol, Satoshi Nambu, and Sunghye Cho. (2018). Prosodic focus of telephone numbers in Tokyo Japanese. Journal of the Acoustical Society of America 143(5). EL340-EL346.*

**Table:** 

**Value of the data** • The data present a mismatch in focus prosody between different positions in digit strings, suggesting that focus prosody can vary within a single language. • The attached csv file (Appendix A1) contains time-normalized raw pitch contours for each digit string for each focus type, separated by speaker and aggregated by all speakers. The data can be useful to better understand the normative picture of prosody and intonation in Tokyo Japanese in general and its phone numbers in particular. Appendix A2 is a z-scored version of Appendix A1 (See Section 2.4). • The attached csv files (Appendix B) include both raw (B1) and normalized (B2) data of the acoustic measurements. The data are valuable for studying speech recognition, speaker variation in prosodic focus, and prosody modeling and also for conducting additional statistical analyses. • In addition to the data that serve as a benchmark for future research on focus prosody, sharing the stimuli makes the experimental framework applicable to studies on focus prosody in other languages. The research project can be extended to international collaborative work as a cross-linguistic project on prosodic focus.

## 1 Data

The data in this article illustrate how prosodic focus is marked within phone number strings in Tokyo Japanese. Figures represent the summaries of data provided in the attached csv files. Detailed interpretation of a subset of the data can be found in Lee et al. [1].

### 1.1 Bipodic template in Tokyo Japanese

**Fig. 1:**
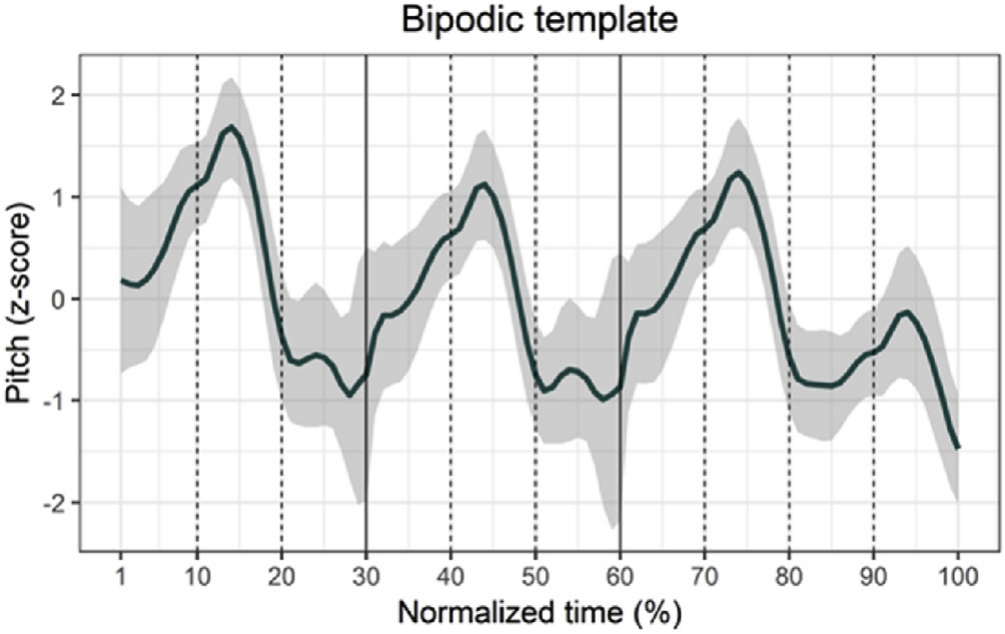
Time-normalized pitch contours of the bipodic template with ten-digit phone number strings in Tokyo Japanese. Dotted lines demarcate each digit and solid lines represent boundaries for each digit group. The area shaded in gray indicates standard deviations of the mean.

Fig. 1 illustrates how phone number strings are realized in Tokyo Japanese, conforming to its particular prosodic structure, known as a bipodic template (Amino and Osanai [2]). The line in Fig. 1 fits a time-normalized pitch contour averaged by 500 ten-digit phone number strings produced for broad focus in the format of (NNN)-(NNN)-(NNNN). In the bipodic template, every two digits join together, and an accentual peak occurs in the second digit. Thus, three-digit strings contain one accentual peak in the second digit, and the four-digit string has two accentual peaks—in the second and fourth digits.

### 1.2 Pitch contours for broad focus and corrective focus

**Fig. 2:**
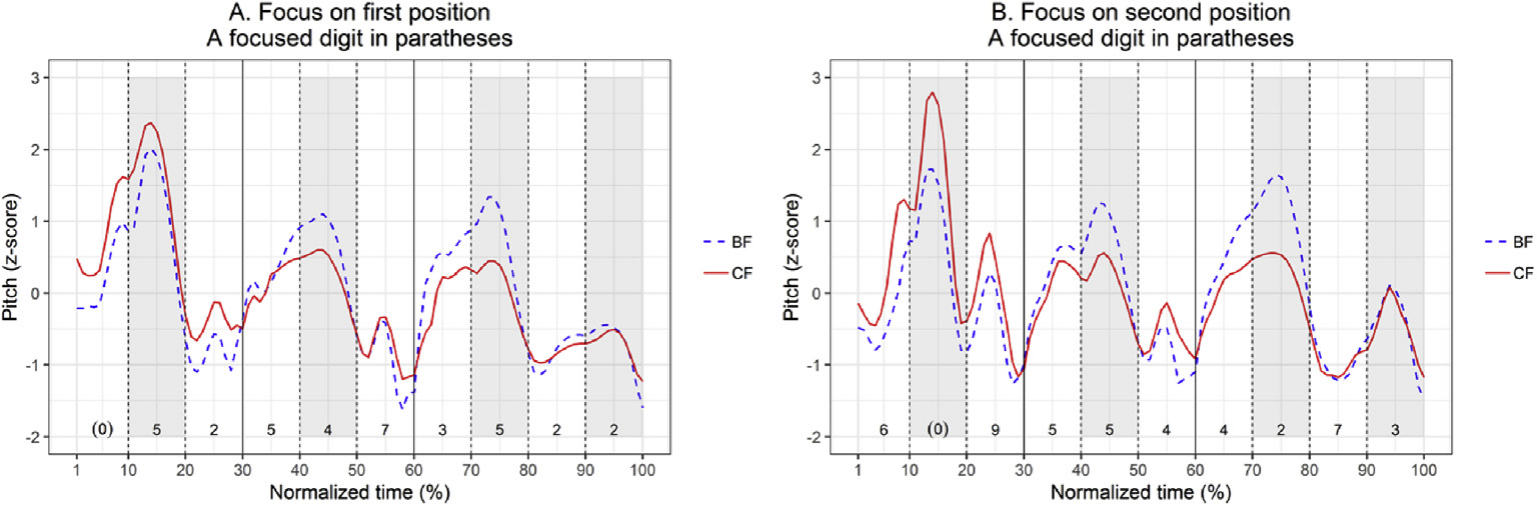
Time-normalized pitch contours: the left panel includes a phone number string with a focused digit in the first position (**N**NN-NNN-NNNN); and the right panel contains a focused digit in the second position (N**N**N-NNN-NNNN). Target focused digits are in parentheses, and the area shaded in gray represents the position containing an accentual peak of each bipodic template.

Fig. 2 shows that focused digits were produced differently depending on their position within the bipodic template. This figure indicates a mismatch in focus prosody between the first and second positions within the bipodic template. The position with an accentual peak is more favorable to focus marking than the position without it. Each line in Fig. 2 is a time-normalized pitch contour averaged by five speakers, in which the red solid line refers to a digit string produced with corrective focus and the blue dotted line shows a digit string under broad focus.

### 1.3 Focus effects in the bipodic template

**Fig. 3:**
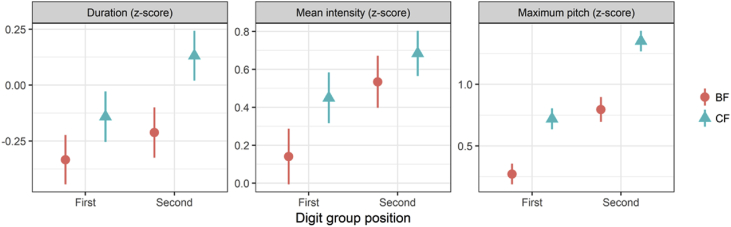
Mean values of duration, mean intensity and maximum pitch as the on-focus effects, separated by focus type (broad vs. corrective) and focus position (first vs. second). BF and CF refer to broad focus and corrective focus, respectively. Error bars indicate 95% confidence intervals.

Fig. 3 demonstrates on-focus effects by duration, mean intensity, and maximum pitch in each of the first and second focus positions within the bipodic template. This figure shows that prosodic effects of focus vary by position within the bipodic template. The points represent the mean values of duration, mean intensity, and max pitch in different focus conditions (broad, corrective), separated by focus positions (first, second) within the bipodic template.

### 1.4 Focus effects by digit position

**Fig. 4:**
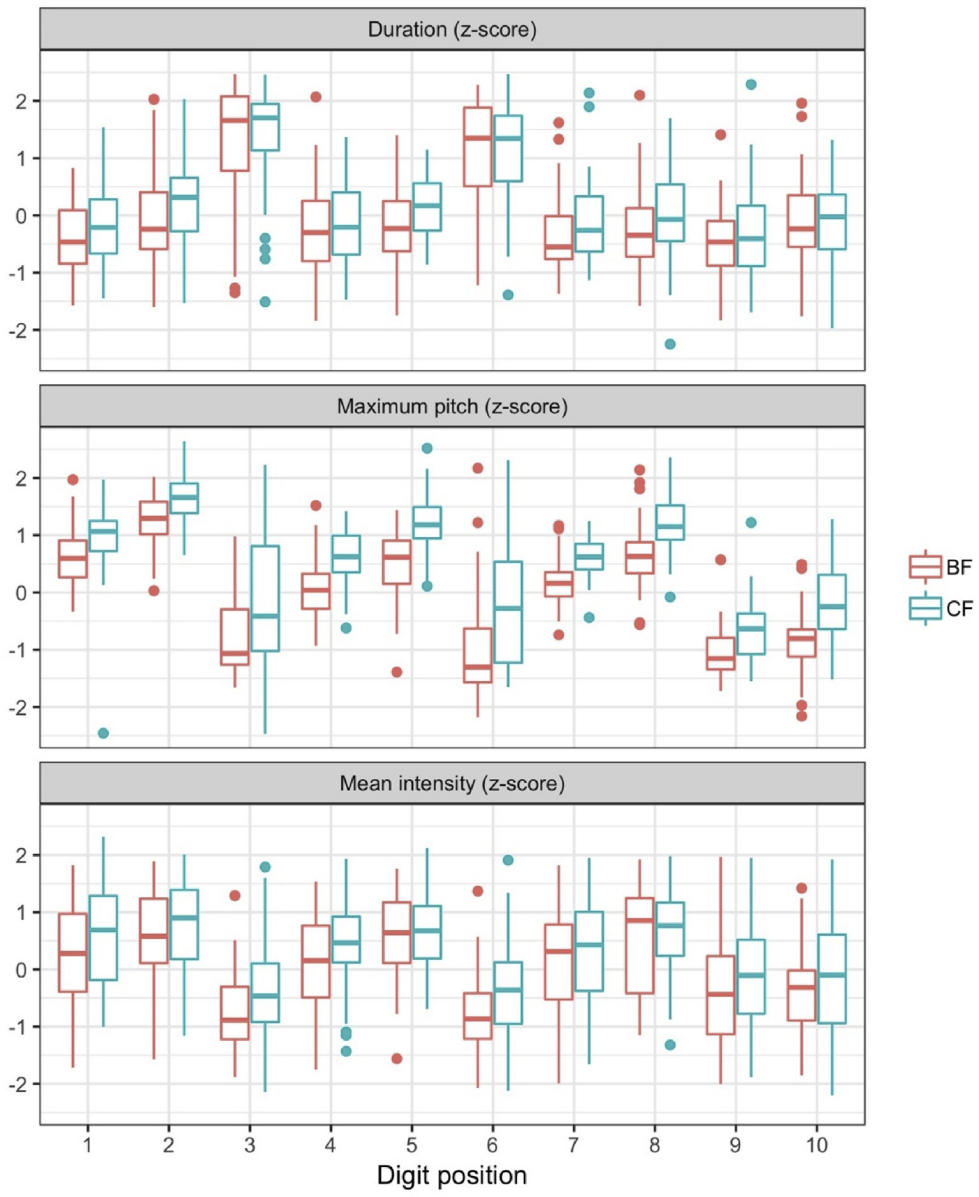
Distributions of duration, maximum pitch, and mean intensity by focus position and focus type. “1” is for the first position of a phone-number string, and “10” is for the last position.

Fig. 4 illustrates z-scores of duration, mean intensity, and maximum pitch in all digit positions within the phone numbers strings, separately for focus type. The on-focus effect is evident in all positions, even though certain positions are more subject to a robust on-focus effect than the other positions due to the bipodic template.

## 2 Experimental design, materials, and methods

### 2.1 Participants

Two male and three females (mean age: 28.4 years), who were naïve to the purpose of the study, participated in the production experiment. They were recruited from the National Institute for Japanese Language and Linguistics in Tokyo and reported history of neither hearing nor speech disorders. All of the speakers were compensated 1,000 yen for their participation after the experiment.

### 2.2 Speech materials

We generated 100 10-digt phone number strings using a Python script (see Appendix C for details). The 100 phone number strings were set up so that each digit (0–9) occurs equally ten times in each position within a digit string and each pair of adjacent digits (e.g., 0–1, 1–2) occurs equally often in each pair of positions within a digit string. We embedded the target stimuli into a question-answer pair to elicit both broad focus and corrective focus (see Appendix D for the entire digit strings). Regarding broad focus, we used a simple yes/no question (1a) where no narrow focus was induced on any particular element of a sentence. For corrective focus, however, a particular question was asked whether a phone number string is correct and the speaker answered by correcting only one incorrect digit in the phone number string, as demonstrated in (1b). In the question-answer pairs, only the answer parts were used to extract acoustic values in the data set.

**Table:** 

(1)	a.	Broad focus
	Q: 番号は分かりましたか	‘Did you get the number?’
	A: はい、番号は367-888-8717です	‘Yes, the number is 367-888-8717.’
	b.	Corrective focus
	Q: 番号は267-888-8717ですか	‘Is the number 267-888-8717?’
	A: いいえ、番号は367-888-8717です	‘No, the number is 367-888-8717.’

Table 1 shows the pronunciation for each digit from 0 to 9 in Tokyo Japanese. Since some digits allow two different pronunciations, the participants were asked to use the forms with an asterisk for consistency in recordings.

**Table 1:** Pronunciations for numerical digits (0–9) in Tokyo Japanese. Pronunciations are transcribed using the Romaji system.

Digit	Pronunciation
0	*zero/rei
1	ichi
2	ni
3	san
4	*yon/shi
5	go
6	roku
7	*nana/shichi
8	hachi
9	kyuu

### 2.3 Recording procedures

Speech materials were recorded using a built-in microphone on a Mac laptop, in a sound-attenuated booth at the National Institute for Japanese Language and Linguistics. Participants sat comfortably before a laptop computer at a distance of about 0.5 m from the microphone. Recordings were conducted at 44.1 kHz sampling frequency and 16-bit resolution, saved directly to the computer as WAV files for acoustic analysis. Speech materials were presented visually to the participants at the center on the computer screen using PowerPoint slides.

There were two recording sessions in the experiment. The first session included stimuli for broad focus, followed by the subsequent session for corrective focus. Prior to the two sessions, a practice session in which they read three 10-digit strings that were irrelevant to the target stimuli was put to let them familiarize with the recording procedure. In the first session, they read sentences for broad focus aloud after listening to a pre-recorded prompt question, as in (1a). After a five-minute break, they read sentences for corrective focus that contain the same sequences as the ones for broad focus but were served in a different context in such a way to correct one digit in a question, as in (1b). The participants were instructed to produce the target stimuli in a natural way, conforming to the design of each session. The experiment yielded a total of 1,000 digit strings (100 digit strings x 5 speakers x 2 focus types).

### 2.4 Acoustic measurements

Using a *Praat* script called ProsodyPro (Xu [3]), we manually marked boundaries for every digit in every digit string. From each labeled interval, we obtained the following three acoustic cues automatically generated from the script: duration in milliseconds, mean intensity in decibels, and maximum pitch in hertz. The script also computed time-normalized pitch contours in hertz at ten equidistant points per each digit in each digit string. The output of this time-normalization for a digit string (Appendix A) is 100 pitch values (= 10 equidistant x 10 digits). Because the broad-focus recordings always preceded the corrective-focus ones in the experiment, the values of duration, mean intensity, maximum pitch, and time-normalized pitch contours were converted to z-scores independently by each speaker and by each digit string in order to counterbalance the order effect and more importantly to normalize inter-speaker variations inherent in acoustic cues for focus marking (Appendix A2 and B2).
