# Supplementary material for: Dataset of focus prosody in Japanese phone numbers
Source: Data Brief. 2019 Jun 11;25:104139. doi: 10.1016/j.dib.2019.104139 (PMC6614727; doi:10.1016/j.dib.2019.104139)
Supplement: Supplementary file 5 [file mmc5.docx]

**Phone number strings in the broad-focus conditions** (underlined)

1. [7]87-412-4699

2. 105-6[0]1-2318

3. 734-5[9]2-8426

4. 011-427-705[0]

5. 366-80[2]-0565

6. [4]46-187-6163

7. 149-665-08[1]0

8. 65[4]-645-3455

9. 759-166-[6]017

10. 499-715-59[6]0

11. 996-[2]09-6296

12. 518-[6]34-9587

13. 861-333-0[9]05

14. 395-8[4]0-1475

15. [3]89-343-4492

16. 0[4]0-776-9911

17. 15[8]-317-5785

18. 076-790-695[6]

19. 579-49[8]-0139

20. 581-994-8[7]04

21. [2]33-653-8838

22. 875-24[6]-5197

23. 84[5]-397-4866

24. 344-142-6[8]06

25. 320-[4]04-7259

26. 25[7]-175-4972

27. 410-616-47[9]0

28. 7[9]1-873-6416

29. 770-1[2]0-5328

30. [1]29-883-2724

31. 648-472-28[5]1

32. [9]47-956-8349

33. 1[8]2-449-3176

34. 471-2[5]2-1382

35. 66[2]-018-3333

36. 6[0]9-554-4273

37. 485-568-[2]071

38. 036-678-154[7]

39. 460-85[9]-0379

40. 298-832-388[9]

41. 065-977-046[1]

42. 543-270-8[6]29

43. 026-988-435[8]

44. 335-084-[5]027

45. 174-214-661[5]

46. 988-[1]03-7578

47. 007-079-793[4]

48. [0]52-547-3522

49. [8]39-221-3208

50. 4[5]0-962-7842

51. 280-506-1[1]14

52. 2[1]3-385-7369

53. 304-32[3]-1895

54. 112-[8]13-3921

55. 853-150-4[5]09

56. 863-70[7]-2648

57. 897-351-[4]094

58. 615-460-[7]146

59. 673-[5]29-8998

60. 5[6]4-763-9236

61. [5]32-757-9745

62. 931-058-[9]184

63. 555-724-[3]083

64. 727-295-[1]032

65. 694-48[0]-0686

66. 822-237-16[7]0

67. 401-582-9[0]07

68. 193-919-2[2]12

69. 819-931-9[3]01

70. 917-[7]38-6393

71. 168-041-855[3]

72. 2[7]8-748-5591

73. 221-7[8]1-1262

74. 241-69[1]-0225

75. 356-510-37[3]0

76. [6]37-686-7664

77. 102-136-[0]041

78. 62[3]-064-2519

79. 784-[0]05-8154

80. 083-896-360[2]

81. 26[9]-099-4123

82. 503-455-98[2]0

83. 4[3]0-289-5631

84. 4[2]4-944-1981

85. 238-[9]28-7437

86. 90[6]-039-1777

87. 314-826-29[4]0

88. 928-5[3]0-9657

89. 592-900-21[0]0

90. 977-8[6]1-5288

91. 525-1[1]1-6535

92. 742-[3]08-8243

93. 686-37[4]-0752

94. 090-369-94[8]0

95. 716-43[5]-2444

96. 967-5[7]1-7713

97. 808-267-[8]067

98. 70[0]-025-6768

99. 95[1]-193-5874

100. 372-622-5[4]03

**Phone number strings in the corrective-focus conditions** (Target digits in square brackets)

1. Q. 887-412-4699 A. [7]87-412-4699

2. Q. 105-611-2318 A. 105-6[0]1-2318

3. Q. 734-502-8426 A.734-5[9]2-8426

4. Q. 011-427-7051 A. 011-427-705[0]

5. Q. 366-803-0565 A.366-80[2]-0565

6. Q. 546-187-6163 A. [4]46-187-6163

7. Q. 149-665-0820 A. 149-665-08[1]0

8. Q. 655-645-3455 A. 65[4]-645-3455

9. Q. 759-166-7017 A. 759-166-[6]017

10. Q. 499-715-5970 A. 499-715-59[6]0

11. Q. 996-309-6296 A. 996-[2]09-6296

12. Q. 518-734-9587 A. 518-[6]34-9587

13. Q. 861-333-0005 A. 861-333-0[9]05

14. Q. 395-850-1475 A. 395-8[4]0-1475

15. Q. 489-343-4492 A. [3]89-343-4492

16. Q. 050-776-9911 A. 0[4]0-776-9911

17. Q. 159-317-5785 A. 15[8]-317-5785

18. Q. 076-790-6957 A. 076-790-695[6]

19. Q. 579-499-0139 A. 579-49[8]-0139

20. Q. 581-994-8804 A. 581-994-8[7]04

21. Q. 333-653-8838 A. [2]33-653-8838

22. Q. 875-247-5197 A. 875-24[6]-5197

23. Q. 846-397-4866 A. 84[5]-397-4866

24. Q. 344-142-6906 A. 344-142-6[8]06

25. Q. 320-504-7259 A. 320-[4]04-7259

26. Q. 258-175-4972 A. 25[7]-175-4972

27. Q. 410-616-4700 A. 410-616-47[9]0

28. Q. 701-873-6416 A. 7[9]1-873-6416

29. Q. 770-130-5328 A. 770-1[2]0-5328

30. Q. 229-883-2724 A. [1]29-883-2724

31. Q. 648-472-2861 A. 648-472-28[5]1

32. Q. 047-956-8349 A. [9]47-956-8349

33. Q. 192-449-3176 A. 1[8]2-449-3176

34. Q. 471-262-1382 A. 471-2[5]2-1382

35. Q. 663-018-3333 A. 66[2]-018-3333

36. Q. 619-554-4273 A. 6[0]9-554-4273

37. Q. 485-568-3071 A. 485-568-[2]071

38. Q. 036-678-1548 A. 036-678-154[7]

39. Q. 460-850-0379 A. 460-85[9]-0379

40. Q. 298-832-3880 A. 298-832-388[9]

41. Q. 065-977-0462 A. 065-977-046[1]

42. Q. 543-270-8729 A. 543-270-8[6]29

43. Q. 026-988-4359 A. 026-988-435[8]

44. Q. 335-084-6027 A. 335-084-[5]027

45. Q. 174-214-6616 A. 174-214-661[5]

46. Q. 988-203-7578 A. 988-[1]03-7578

47. Q. 007-079-7935 A. 007-079-793[4]

48. Q. 152-547-3522 A. [0]52-547-3522

49. Q. 939-221-3208 A. [8]39-221-3208

50. Q. 460-962-7842 A. 4[5]0-962-7842

51. Q. 280-506-1214 A. 280-506-1[1]14

52. Q. 223-385-7369 A. 2[1]3-385-7369

53. Q. 304-324-1895 A. 304-32[3]-1895

54. Q. 112-913-3921 A. 112-[8]13-3921

55. Q. 853-150-4609 A. 853-150-4[5]09

56. Q. 863-708-2648 A. 863-70[7]-2648

57. Q. 897-351-5094 A. 897-351-[4]094

58. Q. 615-460-8146 A. 615-460-[7]146

59. Q. 673-629-8998 A. 673-[5]29-8998

60. Q. 574-763-9236 A. 5[6]4-763-9236

61. Q. 632-757-9745 A. [5]32-757-9745

62. Q. 931-058-0184 A. 931-058-[9]184

63. Q. 555-724-4083 A. 555-724-[3]083

64. Q. 727-295-2032 A. 727-295-[1]032

65. Q. 694-481-0686 A. 694-48[0]-0686

66. Q. 822-237-1680 A. 822-237-16[7]0

67. Q. 401-582-9107 A. 401-582-9[0]07

68. Q. 193-919-2312 A. 193-919-2[2]12

69. Q. 819-931-9401 A. 819-931-9[3]01

70. Q. 917-838-6393 A. 917-[7]38-6393

71. Q. 168-041-8554 A. 168-041-855[3]

72. Q. 288-748-5591 A. 2[7]8-748-5591

73. Q. 221-791-1262 A. 221-7[8]1-1262

74. Q. 241-692-0225 A. 241-69[1]-0225

75. Q. 356-510-3740 A. 356-510-37[3]0

76. Q. 737-686-7664 A. [6]37-686-7664

77. Q. 102-136-1041 A. 102-136-[0]041

78. Q. 624-064-2519 A. 62[3]-064-2519

79. Q. 784-105-8154 A. 784-[0]05-8154

80. Q. 083-896-3603 A. 083-896-360[2]

81. Q. 260-099-4123 A. 26[9]-099-4123

82. Q. 503-455-9830 A. 503-455-98[2]0

83. Q. 440-289-5631 A. 4[3]0-289-5631

84. Q. 434-944-1981 A. 4[2]4-944-1981

85. Q. 238-028-7437 A. 238-[9]28-7437

86. Q. 907-039-1777 A. 90[6]-039-1777

87. Q. 314-826-2950 A. 314-826-29[4]0

88. Q. 928-540-9657 A. 928-5[3]0-9657

89. Q. 592-900-2110 A. 592-900-21[0]0

90. Q. 977-871-5288 A. 977-8[6]1-5288

91. Q. 525-121-6535 A. 525-1[1]1-6535

92. Q. 742-408-8243 A. 742-[3]08-8243

93. Q. 686-375-0752 A. 686-37[4]-0752

94. Q. 090-369-9490 A. 090-369-94[8]0

95. Q. 716-436-2444 A. 716-43[5]-2444

96. Q. 967-581-7713 A. 967-5[7]1-7713

97. Q. 808-267-9067 A. 808-267-[8]067

98. Q. 701-025-6768 A. 70[0]-025-6768

99. Q. 952-193-5874 A. 95[1]-193-5874

100. Q. 372-622-5503 A. 372-622-5[4]03
